# Factors associated with access to HIV testing among international students in Japanese language schools in Tokyo

**DOI:** 10.1371/journal.pone.0235659

**Published:** 2020-07-02

**Authors:** Prakash Shakya, Takashi Sawada, Hong Zhang, Tsutomu Kitajima

**Affiliations:** 1 Department of Community and Global Health, Graduate School of Medicine, The University of Tokyo, Tokyo, Japan; 2 Graduate School of International Co-operation Studies, Kyorin University, Tokyo, Japan; 3 Minatomachi Medical Centre, Yokohama, Japan; University of Toronto, CANADA

## Abstract

Japan has been recognized for its excellent universal health coverage system. However, the migrant population faces many barriers in accessing health services in Japan. Japan hosts around 260,000 international students, mostly from developing countries. Among them, language school students tripled from 2011 to 2017, against the backdrop of labor shortage in Japan. Most of these students are also engaged as cheap laborers and are vulnerable populations with poor access to health services. Several socio-economic and behavioral factors may increase their vulnerability to HIV and prevent them from accessing HIV testing in Japan. We examined the factors associated with access to HIV testing among international students in language schools in Tokyo. We conducted a cross-sectional study among international students studying in Japanese language schools in Tokyo. We collected data from 769 Chinese, Vietnamese, and Nepalese students using a self-administered questionnaire. We measured their access to HIV testing through questions on their knowledge of where to receive HIV testing and utilization of HIV testing. Bivariate and multivariable logistic regression models were used to analyze the data. Nepalese students were less likely to know where to receive HIV testing in Japan than Chinese students (AOR = 0.12, 95% CI 0.01–0.96). Students who did not need Japanese language interpreters during visits to health facilities were more likely to know where to receive HIV testing (AOR = 1.93, 95% CI 1.14–3.25). Students who did not have knowledge of free and anonymous HIV testing in Japan were also less likely to know where to receive HIV testing in Japan (AOR = 0.18, 95% CI 0.08–0.42). Students who did not have knowledge of free and anonymous HIV testing (OR = 0.05, 95% CI 0.02–0.10) and who had not utilized HIV testing in their home country (OR = 0.12, 95% CI 0.06–0.27) were less likely to utilize HIV testing in Japan. Factors associated with access to HIV testing among Japanese language school students in Tokyo are nationality, need for Japanese language interpreters, perceived access to doctors/health workers, utilization of HIV testing in the home country, and knowledge of free and anonymous HIV testing. These findings may help to design interventions for improving access to HIV testing among international students in Japan.

## Introduction

Migration is closely linked to an increase in HIV risk [1]. Social vulnerability in the host country contributes to increased HIV risk among migrants [2–7]. Migrants are frequently separated from their families while also residing in poor living and working conditions. They have poor social support, financial problems, language barriers, and difficulties in adapting to foreign culture and customs [8, 9]. All of these factors may contribute to increased HIV risk [10–12]. Moreover, migrants have poor access to health services, including HIV testing, in the host country [13–15].

Globally, HIV prevalence among migrants is higher than that among the host population [5, 13]. Migrants are also more likely to have a delayed HIV diagnosis [15]. They have specific legal and administrative barriers to access HIV testing, along with cultural and language barriers [13–15]. Most of the studies on access to HIV testing among migrants have been conducted in Europe and North America [13–15]. HIV testing uptake in migrants and ethnic minorities range from 21–73% in US, and 23–64% in Europe [13]. Such studies have also identified several barriers and facilitators for HIV testing, which can be grouped into individual, social, and structural levels. Individual levels consist of demographic, migratory, knowledge, attitudinal, and behavioral factors. Social-level factors indicate the influence of family, friends, and other members within the society. Structural level factors include organizational and policy-related determinants.

Although around 75 million migrants reside in Asia, little is known about their access to healthcare. The socio-cultural composition and diversity in Asian societies is different from that in Western societies. The access to healthcare, including HIV testing, may be different among migrants in Asia. Only a few studies have been conducted among migrants in Japan on their access to healthcare, including HIV testing. Immigration policy in Japan focuses more on control of migrants than on their rights, which may increase the barriers to access to healthcare [16, 17].

In Japan, public health centers known as *Hokenjo* provide free and anonymous HIV testing. The Japanese AIDS Prevention guidelines recommend voluntary counseling and testing for high-risk groups [18]. However, HIV testing in Japan has decreased by 35% from 177,000 in 2008 to 118,000 in 2016 [19]. Further, migrants who are not proficient in Japanese have more difficulty accessing the HIV testing service in Japan. Only a few testing facilities provide interpretation services, in a limited number of languages.

Japan hosts around 2.5 million migrants, of which more than 260,000 are international student migrants [20, 21]. Among them, more than 78,000 are studying in Japanese language schools [20]. Apart from studying in language schools, they have part-time jobs in places such as restaurants, convenience stores, delivering goods, and food packing services. The number of language school students tripled from 2012 to 2017 in the context of Japanese workforce shortage [20]. Around 70% of these students are from developing countries such as China, Vietnam, and Nepal [20]. Most of them are engaged as cheap laborers with poor working and living conditions and are vulnerable populations with poor access to healthcare. Migrants have worse health outcomes in comparison with the Japanese population because of inadequate access to health care [22]. A higher incidence of tuberculosis has been reported among migrants than among the Japanese population due to delayed access to treatment [22]. A study among Nepalese migrants in Japan showed that around 32% of them did not visit the doctor or health worker when needed, which was mostly due to language and health insurance related barriers [23]. Such barriers may also limit migrants’ utilization of HIV testing in Japan. Other barriers may include cultural barriers, financial problems, poor living and working conditions, lack of support system, social stigma, and lack of knowledge about such services. Thus, there is an urgent need to adjust the current system of HIV care services so that they are accessible to international students living in Japan. However, little is known about access to healthcare among migrants in Japan, including their access to HIV testing.

Globally, most of the studies on migrants’ access to HIV care services have focused on labor migrants, not on student migrants. Student migrants are socioeconomically different from labor migrants and are vulnerable to HIV risk. Addressing the factors associated with their access to HIV testing is instrumental in global HIV prevention efforts and improving the overall health status of migrants. In this study, we examined the factors associated with access to HIV testing among international students studying in Japanese language schools in Tokyo.

## Methods

### Study design, area, and participants

We conducted this cross-sectional study in 17 Japanese language schools in Tokyo prefecture, particularly in the Shinjuku ward. Tokyo has the largest number of international students in Japan [21]. We recruited students (both male and female) who fulfilled the following inclusion criteria: 1) Chinese, Vietnamese, or Nepalese citizens, 2) aged 18–49 years, 3) had stayed in Japan for at least 3 months, and 4) were willing to participate voluntarily. We excluded students who could not read and write in Chinese, Vietnamese, or Nepali language.

### Sampling strategy and sample size

We used a convenience sampling method to recruit students. We obtained a list of Japanese language schools registered in the Shinjuku ward office. Of the 38 registered schools, 5 did not have Chinese, Nepalese, or Vietnamese students. Therefore, we contacted or visited 33 Japanese language schools with the help of the Shinjuku health center. Among them, we conducted a self-administered questionnaire survey in the 17 schools that agreed to participate in the survey.

We calculated the required minimum sample size using the G power software version 3.1 for 80% power and alpha error probability of 0.05. For this assumption, we used data from a previous study conducted among Myanmar migrants residing in Thailand [24]. We conducted the two-tailed z-test by entering the assumed data on the difference between two independent proportions (0.07 and 0.02) for the association of host country language skills and HIV testing. The calculated minimum sample size was 538. However, we recruited more participants, considering the possibility of missing data and non-response to the self-administered questionnaire due to the sensitivity of the topic. Consequently, we recruited a total of 769 Chinese, Vietnamese, and Nepalese students.

### Measurements

#### Dependent variable

*Access to HIV testing*. We measured students’ access to HIV testing in Japan by asking a) if they knew where to receive HIV testing in Japan, and b) if they had ever utilized HIV testing in Japan.

#### Independent variables

*Sociodemographic characteristics*. We asked questions about socio-demographic characteristics such as age, gender, nationality, marital status, and educational level obtained in the home country.

*Migration-related characteristics*. We asked questions about their length of stay in Japan, visa status, and type of work. We assessed Japanese language skill using a 7-item questionnaire. It contained questions about conversation, reading, and writing in Japanese. Each item score ranged from zero (not at all) to three (excellent). The total possible scores ranged from zero to 21. This questionnaire has been previously used in another study of migrants in Japan [23, 25]. The Cronbach's alpha coefficient was 0.84 for this study.

*Health*, *behavior*, *and HIV-related characteristics*. We asked questions about their health insurance, alcohol use, need for Japanese language interpreters while visiting health facilities, utilization of HIV testing in their home country and in Japan, knowledge of free and anonymous HIV testing in Japan, and interest in taking HIV tests in Japan. We measured their self-rated health status using a 5-point Likert scale (excellent, very good, good, fair, or poor). This tool has been previously used among migrants in Central Asia [26]. We assessed knowledge about transmission and prevention of HIV using a 12-item questionnaire [27, 28]. The total scores ranged from 12 to 24, with one point for each incorrect response and two points for each correct response. The Cronbach’s alpha coefficient was 0.62 for this study. We assessed perceived risk of HIV using the perceived risk of HIV infection scale (PRHS) [29]. It is an 8-item scale with a total score ranging from 8 to 43. A higher score indicates a higher perceived risk. The Cronbach’s alpha coefficient was 0.68 for this study.

### Data collection

We first translated the questionnaire from English to Chinese, Vietnamese, and Nepali languages. Then, independent translators translated those questionnaires back into English. We finalized it after a series of discussions with expert researchers on migrants’ health and HIV. We collected the data using self-administered questionnaires. We pretested it among 27 students and changed or rephrased a few questions based on the pretest results. We collected the data from September to December 2017. We distributed the questionnaires to the students in the classrooms after explaining the objectives and procedures of the study. The students filled the questionnaires on the spot and returned them to us. We collected the data from a total of 769 students from 17 language schools in Tokyo.

### Data analysis

We conducted descriptive statistics to analyze socio-demographic characteristics, migration-related characteristics, and health, behavior, and HIV-related characteristics. We used chi-squared and Fischer’s exact tests for categorical variables and one-way ANOVA for continuous variables. We used bivariate and multivariable logistic regression models to examine the factors associated with knowledge of where to receive HIV testing in Japan. However, we used only a bivariate logistic regression model to examine the factors associated with utilization of HIV testing in Japan, as the number of students who had ever utilized HIV testing was very low.

All analyses were performed using STATA software, version 14. No multicollinearity was found among the predictor variables. Statistical significance was set at p<0.05.

### Ethical considerations

The Research Ethics Committee of Kyorin University, Tokyo, Japan reviewed and approved this study. The students gave verbal informed consent and participated voluntarily. We did not ask students’ names and assured the students about the confidentiality of their information. We also obtained verbal or written permission from the administration of the language schools to recruit the students.

## Results

### Sociodemographic and migration characteristics

Table 1 compares the socio-demographic and migration characteristics of the students according to their nationalities. Of the total 769 students, 323 were Chinese, 288 were Vietnamese, and 158 were Nepalese. Nepalese students had a mean age of 23.0 years (SD 3.0), compared to 22.0 years (SD 3.0) for Chinese, and Vietnamese students (*p = 0*.*002*). About 54% of Vietnamese students were female, compared to 49% for Nepalese, and 41% for Chinese students (*p = 0*.*01*). About 22% of Nepalese students were married, compared to 3% for Vietnamese, and 2% for Chinese students (*p* = 0.001). The mean length of stay in Japan was 11.7 years (SD 6.4) for Vietnamese students, whereas it was 11.0 years (SD 6.0) for Chinese students, and 10.1 years (SD 6.6) for Nepalese students (*p<0*.*001*). The mean Japanese language skill score was 19.0 (SD 3.7) for Chinese students, whereas it was 15.5 (SD 2.5) for Nepalese students, and 14.8 (SD 3.2) for Vietnamese students (*p<0*.*001*).

**Table 1:** Socio-demographic and migration characteristics of the language school students in Tokyo.

Variables	Total (N = 769)	Chinese (N = 323)		Nepalese (N = 158)		Vietnamese (N = 288)		p-value
		n	%	n	%	n	%	
**Age (years)**	M = 22, SD = 3	M = 22, SD = 3		M = 23, SD = 3		M = 22, SD = 3		0.002
**Gender**								
Male	395	184	57.0	81	51.3	130	45.1	0.01
Female	363	132	40.9	77	48.7	154	53.5	
Others	2	2	0.6	0	0.0	0	0.0	
**Marital status**								
Unmarried	720	310	96.0	133	84.2	277	96.2	<0.001
Married	37	6	1.9	22	13.9	9	3.1	
Others	5	4	1.2	1	0.6	0	0.0	
**Educational level obtained in home country**								
Primary/Secondary	10	6	1.9	4	2.5	0	0.0	
Higher Secondary	444	143	44.3	96	60.8	205	71.2	
Bachelors	271	166	51.4	36	22.8	69	24.0	
Above bachelors	27	2	0.6	18	11.4	7	2.4	
Others	3	0	0.0	0	0.0	3	1.0	
**Length of stay in Japan (months)**	M = 11.1, SD = 6.4	M = 11.0, SD = 6.0		M = 10.1, SD = 6.6		M = 11.7, SD = 6.4		<0.001
**Visa/legal status**								
Student	751	314	97.2	152	96.2	285	99.0	0.099
Dependent	7	2	0.6	4	2.5	1	0.3	
Long term resident	3	2	0.6	1	0.6	0	0.0	
Others	1	1	0.3	0	0.0	0	0.0	
**Type of work in Japan**								
Restaurant	236	63	19.5	49	31.0	124	43.1	<0.001
Convenience store	81	49	15.2	16	10.1	16	5.6	
Bento packing	47	5	1.5	40	25.3	2	0.7	
Factory	38	4	1.2	11	7.0	23	8.0	
Bed making in hotel	48	3	0.9	6	3.8	39	13.5	
No job	200	156	48.3	13	8.2	31	10.8	
Others	94	28	8.7	18	11.4	48	16.7	
**Japanese language skill score**	M = 16.8, SD = 3.9	M = 19.0, SD = 3.7		M = 15.5, SD = 2.5		M = 14.8, SD = 3.2		<0.001

### Health and behavior-related characteristics

Table 2 compares the health and behavior-related characteristics of the students according to their nationalities. About 44% of Chinese students had not consumed alcohol in the last 30 days compared to 69% for Nepalese, and 71% for Vietnamese students (*p<0*.*001*). About 14% of Chinese students self-rated their health status as excellent, compared to 10% for Vietnamese, and 6% for Nepalese students (*p<0*.*001*). About 82% of Nepalese students did not perceive proper access to doctor/health workers, compared to 48% for Chinese, and 32% for Vietnamese students (*p<0*.*001*). About 78% of Nepalese students said that they needed a Japanese language interpreter during visits to health facilities, compared to 74% for Vietnamese, and 53% for Chinese students (*p<0*.*001*).

**Table 2:** Health and behavior-related characteristics of the language school students in Tokyo.

Variables	Total (N = 769)	Chinese (N = 323)		Nepalese (N = 158)		Vietnamese (N = 288)		p-value
	n	n	%	n	%	n	%	
**Alcohol use in last 30 days**								
Everyday	7	5	1.5	0	0	2	0.7	<0.001
2–3 times a week	38	23	7.1	8	5.1	7	2.4	
At least once a week	60	26	8.0	21	13.3	13	4.5	
Less than once a week	203	122	37.8	19	12.0	62	21.5	
Never	450	141	43.7	107	67.7	202	70.1	
**Self-rated health status**								
Excellent	84	46	14.2	10	6.3	28	9.7	<0.001
Very good	254	91	28.2	13	8.2	150	52.1	
Good	172	73	22.6	77	48.7	22	7.6	
Fair	239	101	31.3	55	34.8	83	28.8	
Poor	11	7	2.2	0	0.0	4	1.4	
**Health insurance card**								
Yes	742	309	95.7	150	94.9	283	98.3	0.224
No	22	10	3.1	7	4.4	5	1.7	
**Perceived access to doctor/health worker**								
Yes	387	166	51.4	28	17.7	193	67.0	<0.001
No	367	152	47.1	125	79.1	90	31.3	
**Need of a Japanese language interpreter during visit to health facility**								
Yes	498	170	52.6	119	75.3	209	72.6	<0.001
No	254	148	45.8	33	20.9	73	25.3	

### HIV-related characteristics

Table 3 compares the HIV-related characteristics of the students according to their nationalities. The mean HIV knowledge score for Vietnamese students was 20.3 (SD 1.6), whereas it was 20.0 (SD 2.2) for Nepalese, and 19.9 (SD 2.3) for Chinese students (*p = 0*.*018*). The mean PRHS score for Chinese students was 20.3 (SD 4.5), whereas it was 17.5 (SD 5.4) for Vietnamese, and 14.0 (SD 4.4) for Nepalese students (*p <0*.*001*). About 97% of Nepalese students did not have knowledge of where to receive HIV testing in Japan, compared to 87% for Vietnamese, and 79% for Chinese students (*p <0*.*001*). About 96% of Nepalese students did not have knowledge of free and anonymous HIV testing in Japan, compared to 96% for Vietnamese, and 90% for Chinese students (*p = 0*.*002*). About 36% of Vietnamese students had utilized HIV testing in their home country, compared to 20% for Chinese, and 18% for Nepalese students (*p <0*.*001*). About 6% of Chinese students had utilized HIV testing in Japan, compared to 4% for Vietnamese, and 3% for Nepalese students (*p = 0*.*186*). About 71% of Vietnamese students showed interest in taking HIV tests in Japan in the future, compared to 58% for Nepalese, and 39% for Chinese students (*p <0*.*001*).

**Table 3:** HIV-related characteristics of the language school students in Tokyo.

Variables	Total (N = 769)	Chinese (N = 323)		Nepalese(N = 158)		Vietnamese (N = 288)		p-value
		n	%	n	%	n	%	
**HIV Knowledge score**	M = 20.1, SD = 2.0	M = 19.9, SD = 2.3		M = 20.0, SD = 2.2		M = 20.3, SD = 1.6		0.018
**Perceived risk of HIV (PRHS) score**	M = 18.1, SD = 5.4	M = 20.3, SD = 4.5		M = 14.0, SD = 4.4		M = 17.5, SD = 5.4		<0.001
**Knowledge of where to receive HIV testing in Japan**								
Yes	108	66	20.4	5	3.2	37	12.8	<0.001
No	645	252	78.0	143	90.5	250	86.8	
**Knowledge on free and anonymous HIV testing in Japan**								
Yes	50	33	10.2	6	3.8	11	3.8	0.002
No	704	286	88.5	143	90.5	275	95.5	
**Utilization of HIV testing in home country**								
Yes	192	63	19.5	27	17.1	102	35.4	<0.001
No	558	255	78.9	120	75.9	183	63.5	
**Utilization of HIV testing in Japan**								
Yes	35	20	6.2	5	3.2	10	3.5	0.186
No	716	297	92.0	144	91.1	275	95.5	
**Interest in taking HIV test in Japan**								
Yes	415	125	38.7	85	53.8	205	71.2	<0.001
No	337	194	60.1	61	38.6	82	28.5	

### Factors associated with knowledge of where to receive HIV testing in Japan

Tables 4 and 5 present the bivariate and multivariable logistic regression analysis results of factors associated with knowledge of where to receive HIV testing among language school students in Japan, respectively. Nepalese students were less likely to know where to receive HIV testing in Japan than Chinese students (AOR = 0.12, 95% CI 0.01–0.96). Students who did not have better perceived access to doctors/health workers were less likely to know where to receive HIV testing in Japan (AOR = 0.47, 95% CI 0.28–0.81). Students who did not need Japanese language interpreters during visits to health facilities were more likely to know where to receive HIV testing in Japan (AOR = 1.93, 95% CI 1.14–3.25). Students who did not have knowledge of free and anonymous HIV testing in Japan were also less likely to know where to receive HIV testing in Japan (AOR = 0.18, 95% CI 0.08–0.42). Students who did not have an interest in taking an HIV test in Japan were less likely to know where to receive HIV testing in Japan (AOR = 0.55, 95% CI 0.31–0.97).

**Table 4:** Bivariate analysis for factors associated with knowledge of where to receive HIV testing among the language school students in Tokyo.

Variables	Knowledge of where to receive HIV testing in Japan			
	OR	95% CI		p value
**Age**	0.94	0.87	1.01	0.077
**Gender**				
Male				
Female	0.82	0.54	1.24	0.344
Others	5.50	0.34	89.14	0.230
**Nationality**				
Chinese				
Nepalese	0.13	0.05	0.34	**<0.001**
Vietnamese	0.57	0.36	0.88	**0.011**
**Marital status**				
Unmarried				
Married	0.33	0.08	1.38	0.129
Others	1.43	0.16	12.94	0.749
**Education level obtained in the home country**				
Higher secondary				
Bachelors	0.95	0.61	1.46	0.805
Others	0.68	0.23	1.98	0.480
**Health insurance card**				
Yes				
No	0.28	0.37	2.08	0.212
**Perceived access to doctor/health worker**				
Yes				
No	0.36	0.23	0.57	**<0.001**
**Need of a Japanese language interpreter during visit to health facility**				
Yes				
No	2.37	1.56	3.60	**<0.001**
**Utilization of HIV testing in home country**				
Yes				
No	0.55	0.35	0.84	**0.006**
**Utilization of HIV testing in Japan**				
Yes				
No	0.32	0.15	0.68	**0.003**
**Knowledge on free and anonymous HIV testing in Japan**				
Yes				
No	0.15	0.08	0.27	**<0.001**
**Interest in taking HIV test in Japan**				
Yes				
No	0.68	0.45	1.04	0.076
**Japanese language skill score**	1.08	1.03	1.14	**0.004**
**Perceived risk of HIV (PRHS) score**	1.09	1.05	1.13	**<0.001**

**Table 5:** Multivariable analysis for factors associated with knowledge of where to receive HIV testing among the language school students in Tokyo.

Variables	Knowledge of where to receive HIV testing in Japan (N = 608)			
	AOR	95% CI		p value
**Age**	0.98	0.88	1.10	0.751
**Gender**				
Male				
Female	1.02	0.62	1.68	0.933
Others	1.89	0.04	81.35	0.741
**Nationality**				
Chinese				
Nepalese	0.12	0.01	0.96	**0.046**
Vietnamese	0.78	0.39	1.57	0.484
**Marital status**				
Unmarried				
Married	1.11	0.19	6.44	0.911
Others	0.54	0.02	17.97	0.728
**Education level obtained in the home country**				
Higher secondary				
Bachelors	0.95	0.47	1.90	0.877
Others	0.52	0.10	2.79	0.448
**Health insurance card**				
Yes				
No	0.48	0.05	4.38	0.519
**Perceived access to doctor/health worker**				
Yes				
No	0.47	0.28	0.81	**0.006**
**Need of a Japanese language interpreter during visit to health facility**				
Yes				
No	1.93	1.14	3.25	**0.014**
**Utilization of HIV testing in home country**				
Yes				
No	0.79	0.45	1.38	0.411
**Utilization of HIV testing in Japan**				
Yes				
No	1.12	0.33	3.88	0.854
**Knowledge on free and anonymous HIV testing in Japan**				
Yes				
No	0.18	0.08	0.42	**<0.001**
**Interest in taking HIV test in Japan**				
Yes				
No	0.55	0.31	0.97	**0.038**
**Japanese language skill score**	1.04	0.96	1.12	0.323
**Perceived risk of HIV (PRHS) score**	1.04	0.99	1.10	0.119

### Factors associated with utilization of HIV testing in Japan

Table 6 presents the bivariate logistic regression analysis results of factors associated with utilization of HIV testing among language school students in Japan. Students who did not know where to receive HIV testing in Japan were less likely to utilize HIV testing in Japan (OR = 0.32, 95% CI 0.15–0.68). Students who had not utilized HIV testing in their home country were less likely to utilize HIV testing in Japan (OR = 0.12, 95% CI 0.06–0.27). Students who did not have knowledge of free and anonymous HIV testing in Japan were less likely to utilize HIV testing in Japan (OR = 0.05, 95% CI 0.02–0.10). Students who had higher HIV knowledge scores were less likely to utilize HIV testing in Japan (OR = 0.86, 95% CI 0.74–0.99).

**Table 6:** Bivariate analysis for factors associated with utilization of HIV testing among the language school students in Tokyo.

Variables	Utilization of HIV testing in Japan			
	OR	95% CI		p value
**Age**	1.10	0.99	1.23	0.063
**Gender**				
Male				
Female	0.75	0.37	1.51	0.422
Others	18.35	1.11	304.22	0.042
**Nationality**				
Chinese				
Nepalese	0.52	0.19	1.40	0.194
Vietnamese	0.54	0.25	1.17	0.120
**Marital status**				
Unmarried				
Married	2.82	0.94	8.48	0.065
Others	5.63	0.61	51.95	0.127
**Education level obtained in the home country**				
Higher secondary				
Bachelors	1.14	0.53	2.42	0.742
Others	2.87	0.91	9.02	0.071
**Self-rated health status**				
Excellent/very good/good				
Fair/poor	1.74	0.88	3.44	0.114
**Knowledge of where to receive HIV testing in Japan**				
Yes				
No	0.32	0.15	0.68	**0.003**
**Need of a Japanese language interpreter during visit to health facility**				
Yes				
No	1.06	0.52	2.18	0.876
**Utilization of HIV testing services in home country**				
Yes				
No	0.12	0.06	0.27	**<0.001**
**Knowledge on free and anonymous HIV testing services in Japan**				
Yes				
No	0.05	0.02	0.10	**<0.001**
**Interest in taking HIV test in Japan**				
Yes				
No	0.55	0.27	1.14	0.108
**HIV knowledge score**	0.86	0.74	0.99	0.038
**Perceived risk of HIV (PRHS) score**	1.05	0.98	1.12	0.164

## Discussion

Japan has been recognized for its excellent universal health coverage system, which provides easy access to health care for the Japanese population [30]. However, vulnerable migrant populations, including international students, face many barriers in accessing health services in Japan [30]. This study showed that international students studying in Japanese language schools in Tokyo had poor access to HIV testing in Japan. More than 85% of the students did not know of where to receive HIV testing in Japan. Only 5% of the students had utilized HIV testing in Japan. The factors associated with their knowledge of where to receive HIV testing in Japan were nationality, need for Japanese language interpreters, perceived access to doctors/health workers, knowledge of free and anonymous HIV testing, and interest in taking HIV tests in Japan. The factors associated with their utilization of HIV testing in Japan were knowledge of where to receive HIV testing in Japan, utilization of HIV testing in home country, knowledge of free and anonymous HIV testing, and HIV knowledge score.

In this study, about two-thirds of the students felt the need for a Japanese language interpreter during their visit to a health facility in Japan. They were also less likely to know where to receive HIV testing in Japan. Access to healthcare becomes difficult when there are differences between the languages of healthcare providers and of patients [31]. This leads to various problems such as miscommunication, low compliance, and poor quality of services [32–35]. Health workers have difficulty diagnosing health problems when they are unable to understand the medical history of the patients. Such language barriers also discourage patients from seeking healthcare services, thus resulting in low utilization [35–37]. In Canada, women from HIV-endemic countries had limited access to HIV testing due to English or French language barriers [38]. In a similar population in the US, both men and women were less likely to utilize such services due to poor spoken English proficiency [39]. Thus, one of the key interventions to improve access to HIV testing among migrants is to provide professional interpreter services to those with poor local language skills.

Nepalese students were less likely to know where to receive HIV testing in Japan than were Chinese students. One of the reasons behind this may be the differences in Japanese language proficiency between Nepalese and Chinese students. The mean Japanese language skill score was significantly higher for Chinese students than for Nepalese students. Moreover, a higher percentage of Nepalese students said that they needed a Japanese language interpreter during visits to health facilities than did Chinese students. Another reason may be differences in perceived risk of HIV. Chinese students had a significantly higher perceived risk of HIV than Nepalese students. Studies in the US and UK showed that among Latino and African male migrants, a higher perceived risk of HIV acted as a facilitator for increased use of HIV testing [40–42].

We found that students who did not have better perceived access to doctors/health workers were less likely to know where to receive HIV testing in Japan. Poor access to general health services also limits access to HIV testing. Therefore, it is imperative to improve access to general health services among migrants to increase their HIV testing uptake. A study among Central American women migrants in Texas, USA showed that access to health workers facilitates uptake of HIV testing [43]. In another study in the US, migrants had reduced odds of HIV testing if they had not visited a health worker in the last year [44].

This study showed that knowledge of free and anonymous HIV testing is associated with knowledge of where to receive HIV testing and utilization of HIV testing among students. Stigma associated with HIV further limits the use of HIV testing in a foreign country. Therefore, knowledge of anonymous HIV testing may encourage migrants to use such services. Furthermore, financial hardship makes migrants reluctant to use health services. They are more likely to utilize HIV testing if they know that it is free of cost.

Students who had not utilized HIV testing in their home country were less likely to utilize HIV testing in Japan. Those who had utilized HIV testing in their home country may be more aware and knowledgeable on the importance and benefits of HIV testing, which may encourage them to utilize HIV testing in Japan. It is one of the factors where intervention may be done pre-migration to improve the access to HIV testing in Japan.

Students who had higher HIV knowledge scores were less likely to utilize HIV testing. Those who are aware of HIV transmission and prevention may have less risky sexual behaviors. Therefore, they may have less HIV risk perception and be less likely to utilize HIV testing.

The study results should be interpreted considering the following limitations. Selection bias might have occurred due to convenience sampling. Therefore, the sample may not be representative of other international students in Japanese language schools in Tokyo. Such a selection bias may have resulted in over- or underestimation of the association of factors with access to HIV testing. However, we did not conduct a sensitivity analysis to estimate the magnitude and direction of the selection bias. It may be impractical to obtain representative samples among hard-to-reach populations, such as migrants. Convenience sampling may be the best method to obtain the relevant sample [45].

We did not ask questions about their risky sexual behaviors and history of sexually transmitted diseases. The setting of the data collection was a classroom, which might have made students reluctant to answer about such highly sensitive topics. However, such information may clarify their need to access HIV testing in Japan.

We did not assess the role of stigma in access to HIV testing among the students. Students may not visit health facilities for HIV testing due to HIV-related societal and/or internalized stigma. Future studies should also examine the association of HIV stigma with access to HIV testing.

Despite these limitations, this study is vital as it examined the factors associated with access to HIV testing among one of the less studied but highly vulnerable migrant populations, student migrants. We also compared these factors according to different nationalities, which may be beneficial in designing tailor-made interventions in the future. This is also the pioneer study conducted on access to HIV testing among student migrants of different nationalities residing in Japan.

## Conclusion

Factors associated with access to HIV testing among Japanese language school students in Tokyo are nationality, need for Japanese language interpreters, perceived access to doctors/health workers, utilization of HIV testing in the home country, and knowledge of free and anonymous HIV testing. Most of these factors are modifiable or mutable. Mutable factors are more effective in intervening and useful in promoting access to health care. Thus, these findings may be vital in designing interventions for improving access to HIV testing among international students in Japan. Such interventions include providing language interpreter services in HIV testing facilities and disseminating information about free and anonymous HIV testing among the students.

## Supporting information

S1 File Questionnaire Chinese version. (DOCX)

S2 File Questionnaire English version. (DOCX)

S3 File Questionnaire Nepali version. (DOCX)

S4 File Questionnaire Vietnamese version. (DOCX)
