# Supplementary material for: Factors associated with access to HIV testing among international students in Japanese language schools in Tokyo
Source: PLoS One. 2020 Jul 2;15(7):e0235659. doi: 10.1371/journal.pone.0235659 (PMC7332052; doi:10.1371/journal.pone.0235659)
Supplement: S1 File — (DOCX) [file pone.0235659.s001.docx]

调查问卷（预备调查）

调查关于在东京的日本语学校在校生对于结核病与艾滋病的风险认知、知识以及医疗保健方面的认识。

受访者编号

如题中没有明确要求，请圈出您认为正确的答案编号

例：您认为自己的健康状况怎么样？

1 非常好 2 很好 3 好 4 还可以 5 不好

注：个别问题中有关于对于您在日本过去12个月的提问，如果您在日本的时间少于12个月，请以您在日本的全部时间为基准作答

1.0个人信息

1. 您今年 ___ 岁
2. 您的性别/社会性别是？

1　男性 2 女性 3 其他 ___ （请注明）

1. 您的国籍是？

1 中国 2 尼泊尔 3 越南 4 其他 _____ （国家名称）

1. 您的婚姻状况是？

1 单身 2 已婚 3 其他 ___ （请注明）

1. 您在母国的教育程度是？

1 未就学/非统一教育 2 中小学教育 3 高中教育 4 大学毕业

5 硕士毕业及以上 6 其他___（请注明）

1. 您在日本总共待了几年？ _______年_______个月
2. 您现在的签证种类是？

1 留学签证 2 家族滞在 3 定住者签证 4 永住者签证 5 其他___（请注明）

1. 请问您在日本从事的工作是？（非常勤/常勤）

（如果有多个答案，请回答在过去三个月从事时间最长的工作）

1 餐厅 2 便利店 3 便当店 4 工厂 5 酒店客房服务员 （例如从事铺床等）

6 暂无工作 7 其他___（请注明）

2.0 有关您的语言能力

| 201. 请填写您现在的日语水平 | 一窍不通 | 略知一二 | 良好 | 非常好 |
| --- | --- | --- | --- | --- |
| 1. 日语口语 | 0 | 1 | 2 | 3 |
| 2. 读平假名片假名 | 0 | 1 | 2 | 3 |
| 3. 写平假名片假名 | 0 | 1 | 2 | 3 |
| 4. 读汉字 | 0 | 1 | 2 | 3 |
| 5. 写汉字 | 0 | 1 | 2 | 3 |
| 6. 读日语书或者报纸 | 0 | 1 | 2 | 3 |
| 7. 用日语发电子邮件以及写信 | 0 | 1 | 2 | 3 |
| 请填写您现在的英语水平 |  |  |  |  |
| 1. 英语口语 | 0 | 1 | 2 | 3 |
| 2. 读英语书或报纸 | 0 | 1 | 2 | 3 |
| 3. 用英语发电子邮件以及写信 | 0 | 1 | 2 | 3 |

3.0关于您在日本的生活以及工作

1. 您现在在日本与谁一起生活？

1 朋友 2 家人 3 亲戚 4 独自生活 5 其他

302. 您现在与几个人一起生活（几个人共住一个房间）?

1. 一个人一间房 2. 与 ___ 人共用一间房（请填写人数）

303. 您每周进行有偿劳动的时间大约为多少个小时？　　　______小时

304. 您在日本平均每个月的劳动所得收入是多少?

1. 不满5万日元　 　2. 5万～10万日元 　　3. 10万～20万日元

4. 20万日元以上 　　 5. 没有定期收入

305. 您每日的平均睡眠时间是？

1. 8 小时以上 2. 7-8 小时　　 3. 6-7 小时 4. 6小时不到

4.0 酒精摄入量与自我健康评估

401. 过去30天中您摄取的含酒精饮料的频率为?

1. 每天 2. 每周2-3次 3. 每周1次以上 4. 每周1次不到 5. 不喝含酒精饮料

402. 您认为您现在的健康状况如何？

1. 非常好　 2. 很好 3. 好 4. 一般般 5. 不好

5.0 有关健康保险的信息

501. 您现在持有日本健康保险（持有保险证）吗?

1. 有 2. 没有

502. 您有定期支付您的保险金吗?

1. 每个月或者每两个月支付一次 2. 已有3-6个月未曾支付

3. 已有6-12个月未曾支付 4. 一年以上未曾支付

503. 您认为健康保险对您有用吗?

1. 有用 2. 没用

504. 您认为健康保险是否昂贵?

1. 昂贵 2.并不昂贵

6.0 在日本的医疗途径

601. 假如您有一天生病了，最开始会去哪里？

1.诊所 2. 医院 　 3.当地药店 4. 保健所

5. 在家疗养 6. 其他___（请注明）

602. 现在您认为自己可以准确找到医生或者医疗相关工作者吗?

1. 认为 2. 不认为

603. 至今为止您有在日本的医院/诊所/医生/医疗相关工作者处就诊的经历吗?

1. 有 2. 没有

604. 在过去的12个月里您是否有生过病或是有过健康问题?

1. 是（如果是，有过几次？____次） 2. 否

605. 在过去的12个月中您是否找过医生或者医疗相关工作者咨询医疗方面的问题？

1. 是（如果是，有过几次？____次） 2. 否

1. 在过去的一年中您是否患过常见疾病需要就诊但未就诊？

1. 有 2. 没有

607. 如果您有一天生病了，对您来说要调整去医疗机关就诊的时间困难吗？

1.非常困难 2. 十分困难 3. 困难

4. 还算容易 5.容易 6. 十分简单

608. 您在医院或诊所就诊时需要日语翻译吗?

1. 需要 2. 不需要

609. 在与医生沟通时有没有获得谁的帮助?

1. 自己与医生沟通 2. 专业翻译

3. 医生一方的工作人员 4. 家人

5. 朋友 6. 自己努力去理解

610. 平时在日本有关健康的信息都是从哪里得知的？

1. 朋友 　　2. 老师 3. 家人/亲戚 4. 医疗设施 (保健所、医院等)

5. 网络 6. 报纸　 7.没有信息来源 8.其他 _____ （请注明）

7.0 有关HIV/AIDS的知识

| No. | 问题 | 选项与编号 |
| --- | --- | --- |
| 701 | 请问您有听说过AIDS这种疾病吗？ | 是…………………………..1  否……………………………2  不清楚………………….3 |
| 702 | 请问在您的亲朋好友中有没有感染HIV或者因AIDS而去世的？ | 是…………………………..1  否……………………………2  不清楚………………….3 |
| 703 | 您认为只要在性行为中正确使用安全套就能预防HIV吗？ | 是…………………………..1  否……………………………2  不清楚………………….3 |
| 704 | 您认为看上去十分健康的人也有可能已经感染HIV吗？ | 是…………………………..1  否……………………………2  不清楚………………….3 |
| 705 | 被蚊虫叮咬有没有可能感染HIV？ | 是…………………………..1  否……………………………2  不清楚………………….3 |
| 706 | 与HIV感染者一同用餐会感染HIV吗？ | 是…………………………..1  否……………………………2  不清楚………………….3 |
| 707 | 感染了HIV的孕妇会将HIV病毒传染给胎儿吗？ | 是…………………………..1  否……………………………2  不清楚………………….3 |
| 708 | 感染了HIV的女性会通过母乳传染给婴儿吗？ | 是…………………………..1  否……………………………2  不清楚………………….3 |
| 709 | 避免性行为就可以预防HIV感染吗？ | 是…………………………..1  否……………………………2  不清楚………………….3 |
| 710 | 与HIV感染者牵手会感染HIV病毒吗？ | 是…………………………..1  否……………………………2  不清楚………………….3 |
| 711 | 使用已被使用过的针头与注射器会感染HIV吗？ | 是…………………………..1  否……………………………2  不清楚………………….3 |
| 712 | 接受HIV感染者的输血会感染HIV吗？ | 是…………………………..1  否……………………………2  不清楚………………….3 |

8.0 HIV的风险意识

| No. | 问题 | 编号 |
| --- | --- | --- |
| 801 | 凭直觉您认为自己患HIV的风险有多大？ | 几乎不可能…………………...1  可能性不大 ……………………...2  也许有可能 ………………..3  有可能… ………………...4  非常有可能……………………..5 |
| 802 | 您有没有因可能感染HIV而感到害怕？ | 完全没有………………….…………1  几乎没有……………..................2  有时会……………….….. ……3  时不时会 …………..4  常常会………………………5  一直都会…………………………...6 |
| 803 | 能想象您自己已经感染HIV吗？ | 十分困难…………………………..1  困难…………………………………..2  很简单………………….3  非常简单…………………………..4 |
| 804 | 自己不可能感染HIV  您对这句话怎么看？ | 非常不同意……………………...1  不同意…………………….……….2  应该不同意..…………3  应该同意…….…….4  同意…………………………………..5  非常同意………………………..……6 |
| 805 | 自己对HIV病毒比较脆弱（易受感染）  您对这句话怎么看 | 非常不同意…………………………1  不同意…………………….………….2  应该不同意..…………3  应该同意…….…….4  同意…………………………………..5  非常同意………………………..…6 |
| 806 | 虽然感染的可能性不大，但仍有可能感染  您认同这句话吗？ | 非常不同意……………………….1  不同意…………………….…………2  应该不同意..…………3  应该同意…….…….4  同意……………………………… 5  非常同意………………………..6 |
| 807 | 我感染HIV病毒的可能性为 | 零………………………………….1  几乎为零………………………..2  很小……………………………....3  中等水平……………………… 4  很大……………………………...5  非常大……………………..…6 |
| 808 | 自己感染HIV意味着什么？您有思考过这个问题吗？ | 从未想过…………………....1  很少思考过………………..…2  有时会思考 ……………...3  时常思考……………..………4 |

9.0 有关结核的知识

请在您认为正确的框中画圈

|  |  | 一定正确 (1) | 应该正确 (2) | 应该不对 (3) | 一定不对 (4) |
| --- | --- | --- | --- | --- | --- |
| 901 | 结核不会通过咳嗽、喷嚏、飞沫传染 |  |  |  |  |
| 902 | 与感染结核的人一起工作生活有可能会感染结核 |  |  |  |  |
| 903 | 就算没有跟结核患者频繁接触也有可能感染结核 |  |  |  |  |
| 904 | 无家可归的流浪汉更容易感染结核 |  |  |  |  |
| 905 | 外国移民感染结核的可能性比较低。 |  |  |  |  |
| 906 | 免疫系统没有很好发挥作用就有可能感染结核 |  |  |  |  |
| 907 | 如果感染了HIV/艾滋病毒则更容易感染结核 |  |  |  |  |
| 908 | 治疗结核很困难 |  |  |  |  |
| 909 | 结核菌可能对药物产生耐药性 |  |  |  |  |
| 910 | 结核病可能对肺造成很大伤害 |  |  |  |  |
| 911 | 您不知道身边有谁感染了结核 |  |  |  |  |
| 912 | 结核病是由细菌引起的 |  |  |  |  |
| 913 | 如果不治疗，有可能因结核病死亡 |  |  |  |  |

10.0结核的风险认识

1. 您认为您今后感染结核的可能性有多大？

1. 非常有可能 2. 有可能 3. 一般 4.不可能

11.0 HIV的检查途径

1. 您认为您在日本是否有可行的途径检测HIV?

1. 是 2. 否

2. 您知道日本哪里可以检测HIV?

1. 是(在哪里 ?) 2. 否

3. 无关检测结果，您是否有在母国检测过HIV?

1. 是 2. 否

4. 无关检测结果，您是否有在日本进行过HIV的检测?

1. 是 2. 否

5. 您知道在日本有免费匿名检测HIV的服务吗?

1. 是 2. 否

6. 今后，您对在日本检测HIV有兴趣吗?

1. 是 2. 否

7. 上一题如回答“是" ，对您来说去检测HIV什么是比较重要的? (可多选)

1. 免费 2. 翻译／语言方面的帮助　 3. 距离车站近等交通方便

4. 个人信息的绝对保密 5. 能在周末检查 6. 能在傍晚检查

7. 其他…………….. （请注明）

12.0 诊断与治疗结核的途径

1. 您认为您在日本是否有可行的途径诊断与治疗结核？

1. 是 2. 否

2. 您知道日本哪里可以诊断与治疗结核?

1. 是(在哪里 ?) 2. 否

3. 您知道日本有结核医疗费减免制度吗?

1. 是 2. 否

4. 至今为止您在母国接受过结核的诊断或者治疗吗?

1. 是 2. 否

5. 至今为止您在日本接受过结核的诊断与治疗吗?

1. 是 2. 否

6. 至今为止您有被诊断过患有结核吗?

1. 是 ( a. 在母国 b. 在日本) 2. 否

7. 上一题如回答"是"，请问您为此服用了多久的药物？

………..个月

感谢您参与调查。
