# Supplementary material for: Factors associated with access to HIV testing among international students in Japanese language schools in Tokyo
Source: PLoS One. 2020 Jul 2;15(7):e0235659. doi: 10.1371/journal.pone.0235659 (PMC7332052; doi:10.1371/journal.pone.0235659)
Supplement: S2 File — (DOCX) [file pone.0235659.s002.docx]

**Questionnaire**

**TB and HIV/AIDS related risk perception, knowledge and access to health care among language school students in Tokyo**

Respondent's ID No.

Please circle the appropriate answer, unless otherwise stated.

1. What do you think about your health condition?

1. Excellent 2. Very good 3. Good

1. So-so 5. Not good

**Note:** Some of the questions here are asked about your activities during last 12 months of stay in Japan. If you have stayed less than 12 months, please consider it as the total period of time you have stayed in Japan.

1. **General information**

101. What is your age? ......... Years

102. Please choose your gender.

1. Male 2.Female 3.Others………….. (Please specify)

103. What is your nationality?

1. Chinese 2.Nepali 3.Vietnamese 4.Others………….. (Name of the country)

104. What is your marital status?

1. Unmarried 2. Married 3. Others……….. (Please specify)

105. Please choose the level of education you have completed in your home country (only one)

1. Illiterate/Non-formal 2. Primary/secondary level 3.Higher secondary level

4. Bachelors 5. Above bachelors 6. Others …………. (Please specify)

106. How long have you been in Japan in total? ….......Years ……. Months

107. What is your current visa status in Japan?

1. Student 2. Dependent 3. Long term resident

4. Permanent resident 5. Others…….. (Please specify)

108. What kind of work /where are you doing in Japan? (Part time/full time)

(If you have multiple answers, please choose the one which you have done for longest period in last 3 months)

1. Restaurant 2. Convenience store 3. Bento company 4. Factory

5. Hotel as house keeper e.g. bed making 6.No job 7. Others......... (Please specify)

**2.0 About your language skill**

| 201. Please indicate your current Japanese language skill. |  |  |  |  |
| --- | --- | --- | --- | --- |
| 1. Japanese conversation | 1. Not at all | 2. So-so | 3. Good | 4. Excellent |
| 2. Reading Hiragana and Katakana | 1. Not at all | 2. So-so | 3. Good | 4. Excellent |
| 3. Writing Hiragana and Katakana | 1. Not at all | 2. So-so | 3. Good | 4. Excellent |
| 4. Reading Kanji | 1. Not at all | 2. So-so | 3. Good | 4. Excellent |
| 5. Writing Kanji | 1. Not at all | 2. So-so | 3. Good | 4. Excellent |
| 6. Reading Japanese books/ newspaper | 1. Not at all | 2. So-so | 3. Good | 4. Excellent |
| 7. Writing email/letters in Japanese | 1. Not at all | 2. So-so | 3. Good | 4. Excellent |
| **English language** |  |  |  |  |
| 1. English conversation | 1. Not at all | 2. So-so | 3. Good | 4. Excellent |
| 2. Reading English books/newspaper | 1. Not at all | 2. So-so | 3. Good | 4. Excellent |
| 3. Writing email/letters in English | 1. Not at all | 2. So-so | 3. Good | 4. Excellent |

**3.0 Living and working in Japan**

301. With whom do you live together in Japan now?

1. Friends 2. Family 3. Relative 4. Alone 5. Others……..

302. With how many people do you live/sleep together in one bed room?

1. I have my own bed room 2. I share room with ……. people (number)

303. How many hours are you engaged in paid work per week? ………… hrs

304. How much do you earn per month regular in Japan?

1. Below 50,000 JPY 2. 50,001- 100,000 3. 100,001- 200,000

4. Above 200,001 5. No regular income

305. How many hours per day you sleep usually?

1. More than 8 hours 2. 7-8 hours

3. 6-7 hours 4. Less than 6 hours

**4.0 Alcohol use and self-rated health status**

401. During the last 30 days how often did you have drinks containing alcohol?

1. Everyday 2. 2-3 times a week 3. At least once a week

4. Less than once a week 5. Never

402. How do you rate your current general health status?

1. Excellent 2. Very good 3. Good

4. Fair 5. Poor

**5.0 Information on Health Insurance**

501. Do you have your Japanese health insurance card (Hokensho)?

1. Yes 2. No

502. Do you pay the premium of the health insurance regularly?

1. I pay monthly or once in two months 2. Not paid since 3-6 months

3. Not paid since 6-12 months 4. Not paid for more than a year

503. Do you think health insurance is beneficial to you?

1. Yes 2. No

504. Do you think the cost of health insurance is expensive for you?

1. Yes 2. No

**6.0 Health service access in Japan**

601. Which is the first place you go if you became ill?

1.Clinic 2. Hospital 3. Local pharmacy 4. Public health centre (Hokenjo)

5. Home treatment 6. Others............ (Please specify)

602. Currently, do you think you have proper access to a doctor/health worker in Japan?

1. Yes 2. No

603. Have you ever been to Hospital/Clinic/Doctor/Health workers during your stay in Japan?

1. Yes 2. No

604. Have you been ill/had health problems in past 12 months?

1. Yes (if yes, how many times…..?) 2. No

605. Have you visited a doctor/health worker for medical consultation in past 12 months?

1. Yes (if yes, how many times…..?) 2. No

606. In the past year, did you need to see a doctor/health worker for an general illness/condition but you did not?

1. Yes 2. No

607. How difficult is to manage your time to visit the health facility in Japan, if you became ill?

1.Extremely difficult 2. Very difficult 3. Difficult

4. Fairly easy 5. Easy 6. Very easy

608. Do you need a Japanese language interpreter when visiting a clinic/hospital?

1. Yes 2. No

609. Who helps you communicate with your doctor?

1. I can communicate myself 2. A professional interpret

3. A staff person at your doctor’s office 4. Family member

5. A friend 6. I do my best to understand

7. I have never visited doctor/health worker in Japan

610. From where you usually get the health related information in Japan?

1. Friends 2. Teachers 3. Family/relatives

4. Health facility (e.g. Public health centre, hospital) 5. Internet resources

6. Newspaper 7. No source available 8. Others…………… (Please specify)

**7.0 Knowledge on HIV/AIDS**

| 701 | Have you ever heard of an illness called AIDS? | 1. Yes 2.No 3. Don`t know |
| --- | --- | --- |
| 702 | Do you have a close relative or close friend who is infected with HIV or has died of AIDS? | 1. Yes 2.No 3. Don`t know |
| 703 | Can people protect themselves from HIV by using condom correctly in each sexual contact? | 1. Yes 2.No 3. Don`t know |
| 704 | Do you think a healthy looking person can be infected with HIV? | 1. Yes 2.No 3. Don`t know |
| 705 | Can a person get the HIV from mosquito bite? | 1. Yes 2.No 3. Don`t know |
| 706 | Can a person get HIV by sharing a meal with an HIV infected person? | 1. Yes 2.No 3. Don`t know |
| 707 | Can a pregnant women infected with HIV transmit the virus to her unborn child? | 1. Yes 2.No 3. Don`t know |
| 708 | Can a woman with HIV transmit the virus to her newborn child through breastfeeding? | 1. Yes 2.No 3. Don`t know |
| 709 | Can people protect themselves from HIV by abstaining from sexual intercourse? | 1. Yes 2.No 3. Don`t know |
| 710 | Can a person get HIV by holding on with HIV infected person`s hand? | 1. Yes 2.No 3. Don`t know |
| 711 | Can a person get HIV by using previously used needle/syringe? | 1. Yes 2.No 3. Don`t know |
| 712 | Can blood transfusion from HIV infected person transmit HIV to others? | 1. Yes 2.No 3. Don`t know |

**8.0 Perceived risk of HIV**

| **No.** | **Questions and Filters** | **Coding categories** |
| --- | --- | --- |
| 801 | What is your gut feeling  about how likely you are to get infected with HIV? | Extremely unlikely…………………...1  Very unlikely………………………...2  Somewhat likely……………………..3  Very likely…………………………...4  Extremely likely……………………..5 |
| 802 | I worry about getting  infected with HIV | None of the time………………………1  Rarely…………………………............2  Some of the time………………….…..3  A moderate amount of time…………..4  A lot of the time………………………5  All of the time………………………...6 |
| 803 | Picturing self getting HIV  is something I find: | Very hard to do………………………..1  Hard to do……………………………..2  Easy to do……………………………..3  Very easy to do………………………..4 |
| 804 | I am sure I will NOT get  infected with HIV | Strongly disagree……………………...1  Disagree……………………………….2  Somewhat disagree……………………3  Somewhat agree……………………….4  Agree…………………………………..5  Strongly agree…………………………6 |
| 805 | I feel vulnerable to HIV infection | Strongly disagree……………………....1  Disagree………………………………..2  Somewhat disagree…………………….3  Somewhat agree………………………..4  Agree…………………………………...5  Strongly agree…………………………6 |
| 806 | There is a chance, no matter how small, I could get  HIV | Strongly disagree………………………1  Disagree………………………………..2  Somewhat disagree…………………….3  Somewhat agree………………………..4  Agree…………………………………...5  Strongly agree………………………….6 |
| 807 | I think my chances of getting infected with HIV are: | Zero…………………………………….1  Almost zero…………………………….2  Small…………………………………...3  Moderate…………………………….…4  Large………………………………..….5  Very Large…………………………..…6 |
| 808 | Getting HIV is something  I have | Never thought about…………………....1  Rarely thought about………………..….2  Thought about some of the time……...3  Thought about often……………..……..4 |

| **9.0** | **Knowledge on TB** |  |  |  |  |
| --- | --- | --- | --- | --- | --- |
| 901 | TB cannot be spread by coughing, sneezing or spitting | 1. Definitely true | 2. May be true | 3. May be false | 4. Definitely false |
| 902 | If you live or work with someone who has TB, you can be infected with TB | 1. Definitely true | 2. May be true | 3. May be false | 4. Definitely false |
| 903 | To be infected with TB, you do not need to be exposed to someone who has TB many times. | 1. Definitely true | 2. May be true | 3. May be false | 4. Definitely false |
| 904 | People who are homeless are more likely to get TB | 1. Definitely true | 2. May be true | 3. May be false | 4. Definitely false |
| 905 | Foreign migrants are less likely to be infected with TB | 1. Definitely true | 2. May be true | 3. May be false | 4. Definitely false |
| 906 | If your immune system is not working properly, it is easier to get TB | 1. Definitely true | 2. May be true | 3. May be false | 4. Definitely false |
| 907 | If you have HIV, it is easier to get TB | 1. Definitely true | 2. May be true | 3. May be false | 4. Definitely false |
| 908 | TB is hard to treat | 1. Definitely true | 2. May be true | 3. May be false | 4. Definitely false |
| 909 | TB bacteria can become resistant to the medication, which is used to treat TB | 1. Definitely true | 2. May be true | 3. May be false | 4. Definitely false |
| 910 | TB disease can severely damage a person’s lungs | 1. Definitely true | 2. May be true | 3. May be false | 4. Definitely false |
| 911 | You cannot tell if someone you know has TB disease | 1. Definitely true | 2. May be true | 3. May be false | 4. Definitely false |
| 912 | TB is caused by a germ | 1. Definitely true | 2. May be true | 3. May be false | 4. Definitely false |
| 913 | People can die from TB if it is no treated | 1. Definitely true | 2. May be true | 3. May be false | 4. Definitely false |

**10.0 Perceived risk of TB (single item measure)**

1. How is your chance/risk to acquire TB in future?

1. Very high 2. High 3. Moderate 4.No chance

**11.0 Access to HIV testing**

1. Do you think you have proper access to HIV testing in Japan?

1. Yes 2. No

2. Do you know where to go for HIV testing in Japan?

1. Yes (where…?) 2. No

3. I don’t want to know the result, but have you ever had an HIV test in your home country?

1. Yes 2. No

4. I don’t want to know the result, but have you ever had an HIV test in Japan?

1. Yes 2. No

5. Did you know about free and anonymous HIV testing in Japan?

1. Yes 2. No

6. Are you interested in taking HIV test in Japan in future?

1. Yes 2. No

7. If yes, what is important for you to make HIV test accessible? (Multiple choice)

1. Free of charge 2. Interpreter/language service 3. Easy access from the station 4. Strict privacy 5. Open on weekend 6. Open in the evening 7. Others…………(Please specify)

**12.0 Access to TB diagnosis and treatment services**

1. Do you think you have proper access to TB diagnosis and/or treatment in Japan?

1. Yes 2. No

2. Do you know where to go for TB diagnosis and/or treatment in Japan?

1. Yes (where…?) 2. No

3. Did you know about subsidized treatment of TB in Japan?

1. Yes 2. No

4. Have you ever utilized the TB diagnosis and/or treatment in your home country?

1. Yes 2. No

5. Have you ever utilized the TB diagnosis and/or treatment in Japan?

1. Yes 2. No

6. Have you ever been diagnosed as a TB patient?

1. Yes ( a. In your home country b. In Japan) 2. No

7. If yes, how long you took medicine for it?

………..months

**THANK YOU**
