# Supplementary material for: Factors associated with access to HIV testing among international students in Japanese language schools in Tokyo
Source: PLoS One. 2020 Jul 2;15(7):e0235659. doi: 10.1371/journal.pone.0235659 (PMC7332052; doi:10.1371/journal.pone.0235659)
Supplement: S3 File — (DOCX) [file pone.0235659.s003.docx]

**प्रश्नपत्र**

**टोक्योमा बस्नुहुने भाषा विद्यार्थीहरुको, क्षयरोग (TB) तथा HIV/AIDS संग सम्बन्धित ज्ञान, आफुले महसुस गरेको रोग लाग्ने सम्भावना र स्वास्थ्य सेवाको पहुँच**

ID no. …………….

कृपया सबै प्रश्नहरुको उत्तर दिनु होला। तपाईंलाई सबैभन्दा उपयुक्त लाग्ने उत्तरको नम्बरमा गोलो चिन्ह लगाउनुहोला। "लेख्नुहोस "अथवा "खुलाउनुहोस" लेखिएको ठाउँमा चाहिं आफ्नो उत्तर प्रस्ट रुपमा लेख्नुहोला।

उदाहरणको लागि:

प्रश्न: तपाईंलाई आफ्नो स्वास्थ्य अवस्था कस्तो छ जस्तो लाग्छ?

उत्तरहरु: 1. असाध्यै राम्रो 2. धेरै राम्रो 3. राम्रो 4. ठिकै 5. नराम्रो

**नोट**: यहाँ सोधिएका केहि प्रस्नहरु, तपाईंको बिगत १२ महिना भित्रको जापान बसाईबारे छन्। यदि तपाईं जापान आउनुभएको १२ महिना भएको छैन भने, तपाईं जति अवधि जापान बस्नु भएको छ, त्यहि अवधि लाई आधार मान्नु होला।

1**.0 सामान्य जानकारी**

|  | तपाईं कति वर्षको हुनुभयो? ....................... वर्ष (लेख्नुहोस) |
| --- | --- |
|  | लिङ्ग?  1. पुरुष 　　　　　　　　　　　　2. महिला 　　　　　　　　　　　　　　　3. अन्य ........................(खुलाउनुहोस) |
|  | तपाईं कुन देशको नागरिक हुनुहुन्छ?  1. चिन 2. नेपाल 3.भियतनाम 4. अन्य ……… (देशको नाम) |
|  | तपाईंको बैवाहिक स्थिति?  1. अबिबाहित 2. विवाहित 3. अन्य ........................(खुलाउनुहोस) |
|  | तपाईंले आफ्नो देशमा पुरा गर्नु भएको पढाई ? (कुनै एक मात्र)  1. अशिक्षित/अनौपचारिक शिक्षा 2. प्राथमिक/ माध्यमिक तह 3. उच्च माध्यमिक तह  4. स्नातक तह 5. स्नातक तह भन्दा माथि 6. अन्य .......................... (खुलाउनुहोस) |
|  | तपाईं जापान बस्नुभएको जम्मा कति समय भयो? ...........बर्ष ………..महिना (खुलाउनुहोस) |
| 1. 107 | हाल जापानमा तपाईंको भिसा कस्तो खालको हो?  1. विद्यार्थी भिसा 2. डिपेन्डेन्ट भिसा 3. लामो समयको बसोबास भिसा  4. स्थायी बासिन्दा/ एजुकेन 5. अन्य ........................ (खुलाउनुहोस) |
| 1. 114 | जापानमा कस्तो ठाउँमा/खालको काम (फुलटाइम/पार्टटाइम) गरिरहनुभएको छ? (यदि एक भन्दा बढी काम भएमा, तपाईंले बिगत ३ महिनामा सबैभन्दा लामो समय सम्म गर्नुभएको काममा मात्र गोलो चिन्ह लगाउनुहोला।  1. रेस्टुरेन्टमा 2. कन्भिनीमा 3. बेन्तो कम्पनीमा 4. उत्पादन फ्याक्ट्री  5. होटेलमा हाउस कीपर जस्तै बेड मेकिङ्ग आदि 6. काम गरेको छैन 7. अन्य .............................(प्रस्ट खुलाउनुहोस) |

**2.0 भाषा क्षमता सम्बन्धि जानकारी**

| 201 | हाल तपाईंको भाषा क्षमता कस्तो छ? तपाईंलाइ उपयुक्त लाग्ने उत्तरको नम्बरमा गोलो चिन्ह लगाउनु होला। |  | |  | |  | |  | |
| --- | --- | --- | --- | --- | --- | --- | --- | --- | --- |
| 1 | जापानी भाषामा बार्तालाप | 1. पटक्कै छैन | | 2. ठिकै | | 3. राम्रो | | 4. एकदमै राम्रो | |
| 2 | हिरागाना र काताकाना पढ्न | 1. पटक्कै छैन | | 2. ठिकै | | 3. राम्रो | | 4. एकदमै राम्रो | |
| 3 | हिरागाना र काताकाना लेख्न | 1. पटक्कै छैन | | 2. ठिकै | | 3. राम्रो | | 4. एकदमै राम्रो | |
| 4 | खान्जी पढ्न | 1. पटक्कै छैन | | 2. ठिकै | | 3. राम्रो | | 4. एकदमै राम्रो | |
| 5 | खान्जी लेख्न | 1. पटक्कै छैन | | 2. ठिकै | | 3. राम्रो | | 4. एकदमै राम्रो | |
| 6 | जापानी भाषाको किताब/पत्रपत्रिका पढ्न | 1. पटक्कै छैन | | 2. ठिकै | | 3. राम्रो | | 4. एकदमै राम्रो | |
| 7 | जापानी भाषामा इमेल /चिट्ठी लेख्न | 1. पटक्कै छैन | | 2. ठिकै | | 3. राम्रो | | 4. एकदमै राम्रो | |
|  | **अंग्रेजी भाषा** |  | | | | | | | |
| 1 | अंग्रेजी भाषामा बार्तालाप | | 1. पटक्कै छैन | | 2. ठिकै | | 3. राम्रो | | 4. एकदमै राम्रो |
| 2 | अंग्रेजी भाषाको किताब/ पत्रपत्रिका पढ्न | | 1. पटक्कै छैन | | 2. ठिकै | | 3. राम्रो | | 4. एकदमै राम्रो |
| 3 | अंग्रेजी भाषामा इमेल/ चिट्ठी लेख्न | | 1. पटक्कै छैन | | 2. ठिकै | | 3. राम्रो | | 4. एकदमै राम्रो |

**3.0 जापानको काम तथा बसाइ सम्बन्धि जानकारी**

| 301 | हाल जापानमा को संग बस्नुहुन्छ?  1. साथिहरु 2. परिवार 3. नातेदार  4. एक्लै 5.अन्य…….. |
| --- | --- |
| 302 | हाल तपाईं कति जना व्यक्तिहरुसंग संगै एउटै कोठा मा बस्नुहुन्छ/ सुत्नुहुन्छ?  1. मेरो आफ्नो छुट्टै सुत्ने कोठा छ। 2. म …..…जना व्यक्तिहरु संग कोठा सेयर गर्छु। (संख्या) |
| 303 | हप्तामा कति घण्टा काम गर्नु हुन्छ (पारिश्रमिक पाउने कामका लागि)? ...............................घण्टा |
| 304 | जापानमा एक महिनामा कति कमाउनु हुन्छ?  1. ५०००० येन भन्दा कम 2. ५०००१ देखि १००००० येन  3. १००००१ देखि २००००० येन 4. २००००१ येन भन्दा माथि 5. निश्चित आम्दानी छैन |
| 305 | तपाईं एक दिनमा प्राय कति घण्टा सुत्नु हुन्छ?  1. ८ घण्टा भन्दा बढी 2. ७ देखि ८ घण्टा सम्म  3. ६ देखि ७ घण्टा सम्म 4. ६ घण्टा भन्दा कम |

**4.0 रक्सी सेवन तथा स्वास्थ्य अवस्था सम्बन्धि जानकारी**

| 401 | पछिल्लो ३० दिनमा कतिपटक रक्सी वा रक्सी भएको पेय पदार्थ पिउनु भयो?  1. संधै 2. हप्ताको दुइ तीन पटक 3. हप्ताको एक पटक  4. हप्ताको एक पटक भन्दा कम 5. पिईन |
| --- | --- |
| 402 | तपाईंलाई हालको आफ्नो स्वास्थ्य अवस्था कस्तो छ जस्तो लाग्छ?  1. असाध्यै राम्रो 2. धेरै राम्रो 3. राम्रो  4. ठिकै 5. नराम्रो |

**5.0 स्वास्थ्य बिमा सम्बन्धि जानकारी**

| 501 | तपाईंसंग आफ्नो स्वास्थ्य बिमाको कार्ड (होकेन्शो) छ?  1. छ 2. छैन |
| --- | --- |
| 502 | तपाईंले नियमित रुपमा स्वास्थ्य बिमाको रकम तिर्नुहुन्छ?  1. महिनै पिच्छे अथवा २ महिनामा एक चोटी तिर्छु। 2. ३-६ महिना देखि तिरेको छैन।  3. ६-१२ महिना देखि तिरेको छैन। 4. १ बर्ष भन्दा बढी समय देखि तिरेको छैन। |
| 503 | तपाईंलाइ स्वास्थ बिमा फाईदाजनक छ जस्तो लाग्छ?  1. लाग्छ 2. लाग्दैन |
| 504 | तपाईंलाइ स्वास्थ्य बिमाको लागि तिर्ने रकम महँगो छ जस्तो लाग्छ?  1. लाग्छ 2. लाग्दैन |

**6.0 जापानमा स्वास्थ्य सेवाको पहुँच सम्बन्धि जानकारी**

| 601 | जापानमा बिरामी भएको बेला कुन ठाउँमा पहिला जानुहुन्छ?  1. क्लिनिक 2. अस्पताल 3. औसधि पसल  4. पब्लिक हेल्थ सेन्टर/ होक्केन जो 5. घरायसी उपचार 6. अन्य.................... खुलाउनुहोस |
| --- | --- |
| 602 | अहिलेको अवस्थामा जापानमा तपाईंको डाक्टर/ स्वास्थकर्मीसँग पहुँच छ? (जस्तै जापानमा बिरामी पर्दा सजिलै गरि उपचार पाउन सक्नु)  1. छ 2. छैन |
| 603 | जापानमा कहिल्यै डाक्टर /स्वास्थ्यकर्मीकोमा जानु भएको छ?  1. छ 2. छैन |
| 604 | बितेको १२ महिनामा कुनै प्रकारको स्वास्थ्य समस्या /बिमारी भोग्नुभएको छ?  1. छ ..................... कति पटक? 2. छैन |
| 605 | बितेको १२ महिनामा बिरामी हुँदा जापानमा डाक्टर /स्वास्थ्यकर्मीसंग भेट्नु भएको छ?  1. छ ..................... कति पटक? 2. छैन |
| 606 | बितेको १२ महिनामा बिरामी हुँदा डाक्टर /स्वास्थ्यकर्मीलाइ देखाउनुपर्ने अवस्था हुँदा हुँदै पनि, के तपाईंले उनीहरुलाइ भेट्नु भएको थिएन ?  1. भेटेको थिएँ 2. भेटेको थिईन |
| 607 | तपाईं बिरामी हुँदा स्वास्थ्य उपचार गराउन स्वास्थ्य संस्था /हस्पिटल जानुपरेमा समय मिलाउन कत्तीको गाह्रो हुन्छ?  1. असाध्यै गाह्रो 2. धेरै गाह्रो 3. गाह्रो  4. ठिकै सजिलो 5. सजिलो 6. धेरै सजिलो |
| 608 | तपाईंलाइ हस्पिटल जाँदा जापानी भाषा अनुवादकको आवस्यकता पर्छ?  1. पर्छ 2. पर्दैन |
| 609 | तपाईंलाइ जापानी डाक्टरसंग कुरा गर्न कसले मद्दत गर्नुहुन्छ ?  1. म आफै कुरा गर्न सक्छु 2. ब्याबसायिक भाषा अनुवादकले 3. हस्पिटल/क्लिनिकको स्टाफले  4. मेरो परिवारको सदस्यले 5. साथिले 6. म आफैले बुझ्ने प्रयास गर्छु  7. जापानमा अहिलेसम्म डाक्टर/स्वास्थ्यकर्मीकोमा गएको छैन |
| 610 | जापानमा प्रायगरी स्वास्थ्य संग सम्बन्धित सूचना कहाँ बाट पाउनु हुन्छ ?  1.साथीहरु 2.विद्यालयको सेन्सेइ 3.परिवार /आफन्त 4.स्वास्थ्य संस्था /अस्पताल  5. इन्टरनेट को माध्यम बाट 6.पत्रपत्रिका 7. कुनै सूचना को स्रोत छैन 8.अन्य........................(खुलाउनुहोस) |

**7.0 HIV/AIDS सम्बन्धि ज्ञान तथा धारणा**

| 701 | AIDS भन्ने रोगको बारेमा सुन्नुभएको छ ? | 1.छ 2. छैन 3. थाहा छैन |
| --- | --- | --- |
| 702 | तपाईंको कुनै नजिकको नातेदार अथवा साथिलाइ HIV लागेको वा AIDS लागेर मृत्यु भएको छ? | 1.छ 2. छैन 3. थाहा छैन |
| 703 | के प्रत्येक यौन सम्पर्कमा  ठिक तरिकाले कन्डोम प्रयोग गर्दा HIV बाट बच्न सकिन्छ ? | 1.छ 2. छैन 3. थाहा छैन |
| 704 | के स्वस्थ देखिने मान्छेलाई HIV लागेको हुन सक्छ? | 1.छ 2. छैन 3. थाहा छैन |
| 705 | के लामखुट्टे ले टोकेर HIV को जीवाणु सर्न सक्छ? | 1.छ 2. छैन 3. थाहा छैन |
| 706 | के HIV लागेको व्यक्ति ले खाएको जुठो खानेकुरा खाँदा HIV सर्न सक्छ? | 1.छ 2. छैन 3. थाहा छैन |
| 707 | के HIV लागेको गर्भवती महिलाबाट उनको गर्भमा भएको बच्चा HIV लाइ सर्न सक्छ? | 1.छ 2. छैन 3. थाहा छैन |
| 708 | के HIV लागेको महिलाले बच्चालाइ दुध खुवाउंदा HIV सर्न सक्छ? | 1.छ 2. छैन 3. थाहा छैन |
| 709 | के यौन सम्पर्क नगरेर HIV बाट बच्न सकिन्छ? | 1.छ 2. छैन 3. थाहा छैन |
| 710 | के HIV लागेको व्यक्तिको हात समात्दा HIV सर्न सक्छ? | 1.छ 2. छैन 3. थाहा छैन |
| 711 | के प्रयोग भैसकेको सुई फेरी प्रयोग गर्दा HIV सर्न सक्छ? | 1.छ 2. छैन 3. थाहा छैन |
| 712 | के HIV भएको व्यक्ति बाट रगत लिंदा HIV सर्न सक्छ? | 1.छ 2. छैन 3. थाहा छैन |

**8.0 आफुले महसुस गरेको HIV/AIDS लाग्ने सम्भावना**

| सि. नं | प्रश्नहरु | कोड बिबरण |
| --- | --- | --- |
| 801 | आफुलाई HIVलाग्ने सम्भावना कत्तिको छ जस्तो लाग्छ? | एकदमै सम्भावना  छैन ………………………1  सम्भावना छैन ……………………………...2  अलिकति सम्भावना छ ………………………3  सम्भावना छ ………………………………4  एकदमै सम्भावना छ ………………………...5 |
| 802 | म HIV लाग्न सक्छ भनेर चिन्तित रहन्छु । | कहिले पनि रहन्न ……………………………1  मुस्किल ले रहन्छु …………………………...2  कहिले काहीं रहन्छु ………………………….3  प्राय चिन्तित रहन्छु............................................4  संधै चिन्तित रहन्छु…………………………..5 |
| 803 | आफुलाई HIV लागेको कल्पना गर्नु मेरो लागि : | धेरै गाह्रो हुन्छ………………………………1  गाह्रो हुन्छ …………………………………2  सजिलो छ …………………………...…….3  धेरै सजिलो छ …………………………...…4 |
| 804 | मलाई विस्वास छ मलाई HIV लाग्न सक्दैन | एकदमै असहमत ………………………...…1  असहमत………………………………….2  अलिकति असहमत………………………….3  अलिकति सहमत …………………………...4  सहमत ……………………………….…..5  एकदमै सहमत……………………………..6 |
| 805 | मलाई  HIV लाग्ने सम्भावना छ भनेर महसुस गर्छु। | एकदमै असहमत ………………………...…1  असहमत………………………………….2  अलिकति असहमत………………………….3  अलिकति सहमत …………………………...4  सहमत …………………………………...5  एकदमै सहमत……………………………..6 |
| 806 | थोरै भएपनि मलाई HIV लाग्ने सम्भावना छ | एकदमै असहमत …………………………..1  असहमत……………………...………….2  अलिकति असहमत……………………...….3  अलिकति सहमत …………………....……..4  सहमत …………………………………..5  एकदमै सहमत………………………...…..6 |
| 807 | मलाई लाग्छ, HIV लाग्ने सम्भावना मेरो लागि | शुन्य छ ……………………………….....1  लगभग शुन्य छ……………………………2  सानो छ ………………………………….3  अलिकति छ …………………………...….4  धेरै छ ………………………………….5  एकदमै धेरै छ …………………………....6 |
| 808 | HIV लाग्नु मेरो लागि | कहिले पनि नसोचेको कुरा हो…………………1  मुस्किल ले सोचेको कुरा हो …………………..2  कहिलेकाहीं सोचेको कुरा हो…………………..3  प्राय सोचेको कुरा …………………………..4 |

| **9.0** | **क्षयरोग (TB) सम्बन्धि ज्ञान तथा धारणा** |  |  |  |  |
| --- | --- | --- | --- | --- | --- |
| 901 | TB खोक्दा, हाच्छिउँ गर्दा वा थुक्दा सर्दैन। | 1. पक्का सहि हो | 2. सायद सहि हो | 3. सायद गलत हो | 4. पक्का गलत हो |
| 902 | यदि तपाईं TB लागेको मान्छे संग बस्नुहुन्छ वा काम गर्नुहुन्छ भने तपाईलाई TB लाग्नसक्छ। | 1. पक्का सहि हो | 2. सायद सहि हो | 3. सायद गलत हो | 4. पक्का गलत हो |
| 903 | TB रोग लाग्नको लागि, तपाईंलाई TB रोग लागेको मान्छेसंग धेरै पटक सम्पर्कमा आउनुपर्दैन। | 1. पक्का सहि हो | 2. सायद सहि हो | 3. सायद गलत हो | 4. पक्का गलत हो |
| 904 | घरबार बिहिन ब्यक्ति (होमलेस)लाई TB रोग लाग्ने सम्भावना धेरै हुन्छ। | 1. पक्का सहि हो | 2. सायद सहि हो | 3. सायद गलत हो | 4. पक्का गलत हो |
| 905 | प्रवासमा बस्नेहरुलाई TB रोग लाग्ने सम्भावना कम हुन्छ। | 1. पक्का सहि हो | 2. सायद सहि हो | 3. सायद गलत हो | 4. पक्का गलत हो |
| 906 | यदि तपाईंको शरीरको रोगसंग लड्ने क्षमताले राम्रो संग काम नगरेमा TB बाट सजिलै संक्रमित हुन सकिन्छ। | 1. पक्का सहि हो | 2. सायद सहि हो | 3. सायद गलत हो | 4. पक्का गलत हो |
| 907 | यदि तपाईंलाई HIV छ भने सजिलै TB लाग्न सक्छ। | 1. पक्का सहि हो | 2. सायद सहि हो | 3. सायद गलत हो | 4. पक्का गलत हो |
| 908 | TB रोग निको पार्न गर्न गाह्रो छ। | 1. पक्का सहि हो | 2. सायद सहि हो | 3. सायद गलत हो | 4. पक्का गलत हो |
| 909 | TB रोगका लागि प्रयोग हुने दबाइ, TB रोगका किटाणु संग लड्न नसक्ने वा प्रतिरोधि हुन सक्छन्। | 1. पक्का सहि हो | 2. सायद सहि हो | 3. सायद गलत हो | 4. पक्का गलत हो |
| 910 | TB रोगले तपाईंको फोक्सोलाई गम्भीर क्षति गर्नसक्छ। | 1. पक्का सहि हो | 2. सायद सहि हो | 3. सायद गलत हो | 4. पक्का गलत हो |
| 911 | यदि तपाईंले चिनेको कसैलाई TB रोग लागेको छ भने, तपाईंले भन्न सक्नु हुँदैन। | 1. पक्का सहि हो | 2. सायद सहि हो | 3. सायद गलत हो | 4. पक्का गलत हो |
| 912 | TB रोग किटाणु बाट लाग्छ। | 1. पक्का सहि हो | 2. सायद सहि हो | 3. सायद गलत हो | 4. पक्का गलत हो |
| 913 | यदि उपचार गरेन भने,TB रोग लागेर मान्छे मर्न सक्छ। | 1. पक्का सहि हो | 2. सायद सहि हो | 3. सायद गलत हो | 4. पक्का गलत हो |

**10.0 आफुलाई महसुस भएको TB रोग लाग्ने सम्भावना**

| 1. | तपाईंलाई भविष्यमा TB रोग लाग्ने सम्भावना कत्तिको छ?  1.साह्रै धेरै 2.धेरै 3.ठिक ठिकै 4.सम्भावना छैन |
| --- | --- |

**11.0 HIV टेस्ट सेवाको पहुँच**

| 1. | के तपाईंलाई लाग्छ कि, जापानमा तपाईंको HIV टेस्ट सेवासंग राम्रो पहुँच छ?  1. लाग्छ 2. लाग्दैन |
| --- | --- |
| 2. | तपाईंलाई जापानमा HIV टेस्ट गर्न जाने ठाउँ थाहा छ?  1.छ (कहाँ……………….?) 2.छैन |
| 3. | हामीलाई तपाईंको रिपोर्ट भन्न आवस्यक छैन, तैपनि तपाईंले कहिल्यै आफ्नो देशमा HIV टेस्ट गर्नु भएको छ?  1.छ 2.छैन |
| 4. | हामीलाई तपाईंको रिपोर्ट भन्न आवस्यक छैन, तैपनि तपाईंले कहिल्यै जापानमा HIV टेस्ट गर्नु भएको छ?  1.छ 2.छैन |
| 5. | के तपाईंलाई जापानमा उपलब्ध नि:शुल्क र गोप्य HIV टेस्ट सेवाको बारेमा थाहा छ?  1.छ 2.छैन |
| 6. | के भबिस्यमा तपाईं जापानमा आफ्नो HIV टेस्ट गर्न इच्छुक हुनुहुन्छ?  1.छ 2.छैन |
| 7. | यदि इच्छुक हुनुहुन्छ भने, तल उल्लेख गरिएका सेवाहरु मध्ये कुन चाहिं सेवा HIV टेस्टको सहज पहुँचको लागि महत्वपूर्ण ठान्नु हुन्छ? (एक भन्दा बढी मा चिन्ह लगाउँदा हुन्छ।)  1.निशुल्क सेवा 2.भाषा अनुवादकको सेवा 3.स्टेशनबाट नजिक 4.पूर्ण गोपनियता  5.शनिबार/आइतबार उपलब्ध सेवा 6. साँझ उपलब्ध सेवा 7.अन्य.................... (खुलाउनुहोस) |

**12.0 TB रोगको निदान तथा उपचार सेवाको पहुँच**

| 1. | के तपाईंलाई लाग्छ कि, जापानमा तपाईंको, TB रोगको निदान तथा उपचार सेवासंग राम्रो पहुँच छ?  1. लाग्छ 2. लाग्दैन |
| --- | --- |
| 2. | तपाईंलाई जापानमा TB रोगको निदान तथा उपचार गर्न जाने ठाउँ थाहा छ?  1.छ (कहाँ……………….?) 2.छैन |
| 3. | के तपाईंलाई जापानमा उपलब्ध सहुलियत सहितको TB रोगको उपचारको बारेमा थाहा छ?  1.छ 2.छैन |
| 4. | के तपाईंले कहिल्यै आफ्नो देशमा TB रोगको निदान वा उपचार सेवा लिनुभएको थियो?  1. थियो 2. थिएन |
| 5. | के तपाईंले कहिल्यै जापानमा TB रोगको निदान वा उपचार सेवा लिनुभएको छ?  1.छ 2.छैन |
| 6. | के तपाईंलाई कहिल्यै TB रोग लागेको पुस्टि भएको थियो?  1.थियो (a.आफ्नी देशमा b.जापान मा ) 2. थिएन |
| 7. | यदि थियो भने कति समयसम्म रोग को औषधि खानुभयो?  ……………………..महिना |

**************** **तपाईंलाई धेरै धेरै धन्यवाद छ।** ****************
