# Supplementary material for: Factors associated with access to HIV testing among international students in Japanese language schools in Tokyo
Source: PLoS One. 2020 Jul 2;15(7):e0235659. doi: 10.1371/journal.pone.0235659 (PMC7332052; doi:10.1371/journal.pone.0235659)
Supplement: S4 File — (DOCX) [file pone.0235659.s004.docx]

**Phiếu điều tra**

- Về sự hiểu biết và nhận biết rủi ro của bệnh lao và HIV/AIDS. Cách tiếp cận với các dịch vụ y tế của du học sinh đang học tiếng tại Tokyo (TB and HIV/AIDS related risk perception, knowledge and access to health care among language school students in Tokyo)

|  |  |  |  |  |  |
| --- | --- | --- | --- | --- | --- |

Số thứ tự:

Ngoại trừ những câu hỏi có hưỡng dẫn, hãy khoanh tròn vào câu trả lời thích hợp nhất theo bạn.

Ví dụ: Tình trạng sức khỏe hiện tại của bạn như thế nào?

1. Rất tốt 2. Tốt 3. Khá tốtBình thường 5. Yếu

Lưu ý: Có một số câu hỏi sẽ liên quan đến thời gian sinh sống tại Nhật Bản trong vòng 12 tháng qua. Nếu thời gian cư trú chưa quá12 tháng, hãy trả lời các câu hỏi dựa theo tổng thời gian cư trú của bạn tại Nhật Bản.

**1-0: Thông tin cá nhân**

101. Độ tuổi: ............. tuổi

102. Giới tính:

1. Nam 2. Nữ 3. Khác: ..................... (cụ thể)

103. Quốc tịch:

1. Trung Quốc 2. Nepal 3. Việt Nam 4. Khác: .................... (cụ thể)

104. Tình trạng hôn nhân:

1. Độc thân 2. Kết hôn 3. Khác: ............................. (cụ thể)

105. Quá trình học tập trong nước( chỉ khoanh tròn vào 1 trong những câu trả lời)

1. Không đi học/ Các hình thức học khác

2. Tốt nghiệp tiểu học/ trung học cơ sở

3. Tốt nghiệp trung học phổ thông

4. Tốt nghiệp đại học

5. Tốt nghiệp cao học

6. Khác ...........................................(cụ thể)

106. Tổng thời gian cư trú tại Nhật Bản: ..........năm..........tháng

107. Visa cư trú tại Nhật Bản:

1. Du học 2. Gia đình 3. Định cư

4. Vĩnh trú 5. Khác ...........................(cụ thể)

108. Công việc hiện tại tại Nhật Bản (làm thêm /chính thức)

(Nếu như có nhiều đáp án, hãy chọn công việc mà bạn gắn bó lâu nhất trong vòng 3 tháng qua)

1. Công việc tại nhà hàng

2. Công việc tại cửa hàng tiện ích

3. Công việc tại cửa hàng bán cơm hộp

4. Công việc tại nhà máy sản xuất

5. Công việc tại khách sạn (vd: dọn phòng)

6. Chưa từng đi làm

7. Khác .....................................(cụ thể)

**2.0: Khả năng ngôn ngữ**

| 201. Hãy chọn câu trả lời thích hợp với khả năng ngôn ngữ hiện tại của bạn |  |  |  |  |
| --- | --- | --- | --- | --- |
| **Tiếng Nhật** |  |  |  |  |
| 1. Hội thoại bằng tiếng Nhật | 1. Yếu | 2. Bình thường | 3. Khá | 4. Tốt |
| 2. Đọc được Hiragana, Katakana | 1. Yếu | 2. Bình thường | 3. Khá | 4. Tốt |
| 3. Viết được Hiragana, Katakana | 1. Yếu | 2. Bình thường | 3. Khá | 4. Tốt |
| 4. Đọc được chữ Hán (Kanji) | 1. Yếu | 2. Bình thường | 3. Khá | 4. Tốt |
| 5. Viết được chữ Hán (Kanji) | 1. Yếu | 2. Bình thường | 3. Khá | 4. Tốt |
| 6. Đọc được sách, báo tiếng Nhật | 1. Yếu | 2. Bình thường | 3. Khá | 4. Tốt |
| 7. Viết được thư hoặc e-mail bằng tiếng Nhật | 1. Yếu | 2. Bình thường | 3. Khá | 4. Tốt |
| **Tiếng Anh** |  | | | |
| 1. Hội thoại bằng tiếng Anh | 1. Yếu | 2. Bình thường | 3. Khá | 4. Tốt |
| 2. Đọc được sách, báo tiếng Anh | 1. Yếu | 2. Bình thường | 3. Khá | 4. Tốt |
| 3. Viết được thư hoặc e-mail bằng tiếng Anh | 1. Yếu | 2. Bình thường | 3. Khá | 4. Tốt |

**3.0: Công việc và cuộc sống tại Nhật Bản**

301. Hiện tại bạn đang sống cùng với ai?

1. Bạn bè 2. Gia đình 3. Họ hàng

4, Một mình 5. Khác..............

302. Bạn ngủ với mấy người trong 1 phòng?

1. Một mình 2. Cùng với......... người

303. Trong 1 tuần bạn làm việc mấy tiếng?................... tiếng

304.Lương tháng của bạn là bao nhiêu?

1. Dưới 50,000 yên 2. 50,001-100,000 yên 3. 100,001-200,000 yên

4. Trên 200,001 yên 5. Không có thu nhập định kỳ

305. 1 ngày bạn ngủ khoảng mấy tiếng?

1. Trên 8 tiếng 2. 7-8 tiếng

3. 6-7 tiếng 4. Dưới 6 tiếng

**4.0: Mức độ sử dụng các dung dịch có cồn và nhận thức về sức khỏe của bản thân**

401. Mức độ uống rượu (hoặc đồ uống có cồn) của bạn trong vòng 30 ngày vừa qua như thế nào?

1. Hằng ngày 2. 2-3 lần trong tuần 3. Nhiều hơn 1 lần trong tuần

4. Ít hơn 1 lần trong tuần 5. Không uống

402. Tình trạng sức khỏe hiện tại của bạn như thế nào?

1. Rất tốt 2. Tốt 3. Khá

4.Bình thường 5. Yếu

**5.0: Thông tin về bảo hiểm y tế**

501. Bạn có bảo hiểm y tế củaNhật Bản (保険証-hokensho) không?

1. Có 2. Không

502.Bạn có đóng tiền bảo hiểm y tế định kì không?

1. Hằng tháng hoặc 2 tháng 1 lần 2. Chưa đóng tiền 3-6 tháng nay

3. Chưa đóng tiền 6-12 tháng nay 4. Chưa đóng tiền 1 năm nay

503. Bạn có nghĩ bảo hiểm y tế có tác dụng không?

1. Có 2. Không

504. Bạn có nghĩ chi phí cho bảo hiểm y tế cao không?

1. Có 2. Không

**6.0: Cách tiếp cận với y tế tại Nhật Bản**

601. Khi ốm hoặc mắc bệnh, bạn thường đến đâu đầu tiên?

1. Phòng khám

2. Bệnh viện

3. Cửa hàng dược phẩm gần nhà

4. Trung tâm y tế của quận

5. Điều trị tại nhà

6. Khác ....................................................(cụ thể)

602. Hiện tại, bạn có nghĩ mình đang được tiếp cận với y tế một cách chính xác?

1. Có 2. Không

603.Bạn đã từng đến khám bệnh tại bệnh viện hoặc trung tâm y tế khi đang cư trú tại Nhật Bản chưa?

1. Đã 2. Chưa

604. Bạn có mắc bệnh hoặc gặp các vấn đề liên quan đến sức khỏe trong vòng 12 tháng qua không?

1. Có (số lần...........) 2. Không

605. Bạn có bao giờ đến nhờ tư vấn của bác sĩ hoặc các chuyên gia y tế về sức khỏe trong vòng 12 tháng qua không?

1. Có (số lần..........) 2. Không

606. Trong năm nay, bạn có bị mắc bệnh mà lại không đi bệnh viện không?

1. Có2. Không

607.Việc sắp xếp thời gian đi bệnh viên khi bạn bị ốm khó như thế nào?

1. Vô cùng khó 2. Khá khó 3. Khó

4. Khá đơn giản 5. Đơn giản 6. Rất đơn giản

608. Lúc đi khám tại bệnh viện hoặc phòng khám ở Nhật Bản, bạn có cần người hỗ trợ về ngôn ngữ không ?

1. Có 2. Không

609. Ai hỗ trợ bạn về ngôn ngữ khi nói chuyện với bác sĩ?

1. Tự nói chuyện 2. Người phiên dịch

3. Nhân viên của bệnh viện 4. Gia đình, người thân

5. Bạn bè 6.Tôi cố gắng hết sức để hiểu

7. Tôi chưa từng gặp bác sĩ hay nhân viên y tế nào tại Nhật

610. Thông thường, bạn lấy được các thông tin liên quan đến sức khỏe từ đâu?

1. Bạn bè 2. Giáo viên 3. Gia đình/ người thân

4. Các cơ quan y tế (vd: bệnh viện, trung tâm y tế) 5. Internet

6. Báo chí 7. Không từ nguồn nào 8. Khác .......................(cụ thể)

**7.0: Kiến thức liên quan đến HIV/AIDS**

| Số | Câu hỏi | Trả lời |
| --- | --- | --- |
| 701 | Bạn có bao giờ nghe hoặc biết đến HIV/AIDS? | 1. Có 2. Không 3. Không rõ |
| 702 | Bạn có người quen hoặc bạn bè đã từng nhiễm HIV hoặc chết vì AIDS không? | 1. Có 2. Không 3. Không rõ |
| 703 | Bạn có nghĩ rằng việc sử dụng bao cao su một cách chính xáccó thể bảo vệ bản thânkhỏi HIV không? | 1. Có 2. Không 3. Không rõ |
| 704 | Bạn có nghĩ là một người khỏe mạnh cũng có nguy cơ nhiễm HIV không? | 1. Có 2. Không 3. Không rõ |
| 705 | Bạn nghĩ sao về khả năng nhiễm HIV khi bị muỗi đốt ? | 1. Có 2. Không 3. Không rõ |
| 706 | Bạn nghĩ sao về khả năng nhiễm HIV khi dùng chung bữa ăn với người mắc bệnh HIV? | 1. Có 2. Không 3. Không rõ |
| 707 | Phụ nữ nhiễm HIV khi mang thai có khả năng lây truyền HIV cho con không? | 1. Có 2. Không 3. Không rõ |
| 708 | Phụ nữ nhiễm HIV có khả năng lây truyền HIV cho con qua đườngsữa mẹ không? | 1. Có 2. Không 3. Không rõ |
| 709 | Bạn có thể tự bảo vệ mình khỏi nhiễm HIV bằng việc tránh các hoạt động tình dục không? | 1. Có 2. Không 3. Không rõ |
| 710 | Bạn nghĩ sao về khả năng nhiễm HIV khi nắm tay với một người mắc bệnh HIV? | 1. Có 2. Không 3. Không rõ |
| 711 | Bạn nghĩ sao về khả năng nhiễm HIV khi sử dụng kim tiêm và ống tiêm đã qua sử dụng? | 1. Có 2. Không 3. Không rõ |
| 712 | Việc tiếp máu từ người mắc bệnh HIV có khả năng gây nhiễm HIV không? | 1. Có 2. Không 3. Không rõ |

**8.0: Nhận thức về nguy cơ nhiễm HIV**

| Số tt | Câu hỏi | Trả lời |
| --- | --- | --- |
| 801 | Bạn cảm thấy thế nào về nguy cơ nhiễm HIV của bản thân mình? | Khá thấp.............................1  Thấp...................................2  Binh thường....................... 3  Cao.................................... 4  Khá cao..............................5 |
| 802 | Bạn có thường cảm thấy lo lắng rằng mình có thể bị nhiễm HIV không? | Hoàn toàn không có...........1  Hiếm khi............................. 2  Đôi khi................................ 3  Thi thoảng.......................... 4  Thường xuyên....................5  Luôn luôn............................6 |
| 803 | Việc tưởng tượng bản thân bạn nhiễm HIV là. | Rất khó...............................1  Khó.....................................2  Dễ.......................................3  Rất dễ.................................4 |
| 804 | Bạn có tin chắc rằng mình sẽ không nhiễm HIV? | Hoàn toàn không đồng ý.....1  Không đồng ý......................2  Không đồng ý 1 phần..........3  Đồng ý một phần.................4  Đồng ý.................................5  Hoàn toàn đồng ý................6 |
| 805 | Bạn có cảm thấy mình dễ bị lây nhiễm HIV không? | Hoàn toàn không đồng ý.....1  Không đồng ý......................2  Không đồng ý 1 phần..........3  Đồng ý một phần.................4  Đồng ý.................................5  Hoàn toàn đồng ý................6 |
| 806 | Mặc dù rất thấp nhưng bạn có nghĩ là mình có khả năng bị nhiễm HIV không? | Hoàn toàn không đồng ý.....1  Không đồng ý......................2  Không đồng ý 1 phần..........3  Đồng ý một phần.................4  Đồng ý.................................5  Hoàn toàn đồng ý................6 |
| 807 | Bạn nghĩ thế nào về khả năng nhiễm HIV của bản thân? | Hoàn toàn không thể...........1  Không thể............................2  Rất ít...................................3  Có khả năng. ...... ...... .......4  Có khả năng cao.................5  Hoàn toàn có thể.................6 |
| 808 | Bạn có thường suy nghĩ đến việc bản thân bị nhiễm HIV bao giờ chưa? | Chưa bao giờ......................1  Ít khi.....................................2  Thi thoảng............................3  Thường xuyên.....................4 |

**9.0: Kiến thức liên quan đến TB ( bệnh lao)**

|  |  |  |  |  |  |
| --- | --- | --- | --- | --- | --- |
| 901 | Bệnh lao không lây lan qua ho, hắt hơi và nước bọt. | 1. Hoàn toàn đúng | 2. Đa phần đúng | 3. Đa phần sai | 4. Hoàn toàn sai |
| 902 | Nếu bạn sống hoặc làm việc cùng người bị bệnh lao, bản thân bạn cũng có thể bị mắc bệnh lao. | 1. Hoàn toàn đúng | 2. Đa phần đúng | 3. Đa phần sai | 4. Hoàn toàn sai |
| 903 | Bạn có thể bị bệnh lao, ngay cả khi bạn tiếp xúc với người bị bệnh lao chỉ vài lần. | 1. Hoàn toàn đúng | 2. Đa phần đúng | 3. Đa phần sai | 4. Hoàn toàn sai |
| 904 | Khả năng mắc bệnh lao của người vô gia cư là khá cao. | 1. Hoàn toàn đúng | 2. Đa phần đúng | 3. Đa phần sai | 4. Hoàn toàn sai |
| 905 | Khả năng mắc bệnh lao của người nhập cư từ nước ngoài là khá thấp. | 1. Hoàn toàn đúng | 2. Đa phần đúng | 3. Đa phần sai | 4. Hoàn toàn sai |
| 906 | Nếu hệ miễn dịch không tốt thì khả năng mắc bệnh lao là rất cao. | 1. Hoàn toàn đúng | 2. Đa phần đúng | 3. Đa phần sai | 4. Hoàn toàn sai |
| 907 | Những người nhiễm HIV/AIDS dễ mắc bệnh lao. | 1. Hoàn toàn đúng | 2. Đa phần đúng | 3. Đa phần sai | 4. Hoàn toàn sai |
| 908 | Bệnh lao rất khó chữa. | 1. Hoàn toàn đúng | 2. Đa phần đúng | 3. Đa phần sai | 4. Hoàn toàn sai |
| 909 | Vị trùng lao có khả năng đối kháng lại với thuốc dùng để trị liệu bệnh lao. | 1. Hoàn toàn đúng | 2. Đa phần đúng | 3. Đa phần sai | 4. Hoàn toàn sai |
| 910 | Bệnh lao có thể gây tổn hại nghiêm trọng đến phổi. | 1. Hoàn toàn đúng | 2. Đa phần đúng | 3. Đa phần sai | 4. Hoàn toàn sai |
| 911 | Rất khó để biết người xung quanh bạn có mắc bệnh lao hay không. | 1. Hoàn toàn đúng | 2. Đa phần đúng | 3. Đa phần sai | 4. Hoàn toàn sai |
| 912 | Bệnh lao xảy ra là do vi khuẩn. | 1. Hoàn toàn đúng | 2. Đa phần đúng | 3. Đa phần sai | 4. Hoàn toàn sai |
| 913 | Nếu không chữa trị kịp thời bệnh lao thì khả năng dẫn đến tử vong là rất cao. | 1. Hoàn toàn đúng | 2. Đa phần đúng | 3. Đa phần sai | 4. Hoàn toàn sai |

**10.0: Nhận thức về nguy hiểm của TB (bệnh lao)**

1. Bạn nghĩ sao về khả năng mắc bệnh lao của bản thân trong tương lai?

1. Rất cao 2. Cao 3. Thấp 4. Hoàn toàn không

**11.0: Cách tiếp cận với các dịch vụ xét nghiệm HIV**

1. Bạn có nghĩ là mình có thể tiếp cận với các dịch vụ xét nghiệm HIV tại Nhật Bản hay không?

1. Có 2. Không

2. Bạn có biết nơi nào ở Nhật Bản có thể giúp bạn về việc xét nghiệm HIV không?

1. Có (cụ thể.......................) 2. Không

3. Bạn đã bao giờ đi xét nghiệm HIV tại nước bạn sinh ra chưa?

1. Có 2. Chưa

4. Bạn đã bao giờ đi xét nghiệm HIV tại Nhật Bản chưa?

1. Có 2. Chưa

5. Bạn có biết về dịch vụ xét nghiệm HIV giấu tên miễn phí ở Nhật không?

1. Có 2. Không

6. Trong tương lai bạn có muốn thử xét nghiệm HIV tại Nhật Bản không?

1. Có 2. Không

7. Trong trường hợp bạn chọn Có ở câu số 6, đối với bạn thì điều gì là quan trọng nhất để dễ dàng làm được xét nghiệm HIV? (nhiều lựa chọn)

1. Miễn phí 2. Phiên dịch/hỗ trợ về ngôn ngữ3.Trung tâm xét nghiện ở gần ga

4. Thông tin cá nhân được bảo mật 5. Trung tâm xét nghiệm mở cửa cuối tuần

6. Trung tâm mở cửa vào buổi tối 7. Khác.........................................(cụ thể)

**12.0: Cách tiếp cận các dịch vụ chẩn đoán và diều trị bệnh lao**

1. Bạn có nghĩ là mình có thể tiếp cận với các dịch vụ chẩn đoán và/hoặc điều trị bệnh lao tại Nhật Bản không?

1. Có 2. Không

2. Bạn có biết nơi nào ở Nhật Bản có thể giúp bạn về việc chẩn đoán và/hoặc điều trị bệnh lao không?

1. Có (cụ thể..............................) 2. Không

3.Bạn có biết về giảm/ miễn chi phí y tế cho bệnh lao ở Nhật Bản không?

1. Có 2. Không

4. Bạn đã bao giờ thử các dịch vụ về chẩn đoán/ điều trị bệnh lao tại nước bạn sinh ra chưa?

1. Có 2. Chưa

5. Bạn đã bao giờ thử các dịch vụ về chẩn đoán/ điều trị bệnh lao tại Nhật Bản chưa?

1. Có 2. Chưa

6. Bạn đã bao giờ được chẩn đoán mắc bệnh lao chưa?

1. Có (a.tại nước bạn sinh ra b.tại Nhật Bản) 2. Chưa

7. Trong trường hợp bạn chọn Có ở câu số 6, bạn đã phải uống thuốc trong bao lâu?

............................ tháng.

**Rất cảm ơn vì sự hợp tác của bạn.**
